# Exploring Family Interactions: Heterogeneity in Family Specific Network Models

**DOI:** 10.1111/famp.70208

**Published:** 2026-10-09

**Authors:** Lea Schumacher, Daniela Hartmann, Felix Vogel

**Affiliations:** ^1^ Department of Child and Adolescent Psychotherapy University of Hamburg Hamburg Germany; ^2^ Department of Clinical Child and Adolescent Psychology University of Giessen Giessen Germany; ^3^ Department of Clinical Psychology and Child and Psychotherapy of Childhood and Adolescence Johannes Gutenberg University Mainz Germany

**Keywords:** family interactions, heterogeneity, idiographic research, network analysis, parental self‐efficacy

## Abstract

Daily family interactions have a profound impact on children's emotional and behavioral development. While extensive research highlights the detrimental effects of negative parenting practices and the benefits of positive parenting programs, less is known about the complex within‐family interactions that occur on a day‐to‐day basis. Using 5 weeks of daily diary data from 43 mothers (*M*_Observations per dyad_ = 140.44, SD = 1.05) aged 33–53 years and their children aged 6–14 years, this paper explored the heterogeneity of family‐specific networks and their links to parental self‐efficacy (PSE) as part of a german national study. Using group iterative multiple model estimation, we identified both contemporaneous and temporal relationships between daily fluctuations in conflicts, praise, quality time, and parental stress. Our results revealed substantial variability in network structures across families. There was no link between network structures and PSE. The current findings indicate that family‐specific network modeling may facilitate the identification of interaction patterns that maintain dysfunctional family dynamics, thereby enabling targeted selection of intervention components. Such personalized approaches could improve intervention efficiency and acceptability through alignment with families' unique regulatory processes.

## 1 Introduction

Daily family interactions as moment‐to‐moment exchanges between parents and children in everyday life play a central role in children's emotional and behavioral development (Johnson and Ray 2016). These interactions often unfold as recurring patterns in which the behavior and emotional states of family members influence one another (Rönkä and Korvela 2009). For example, heightened parental stress may increase parent–child conflict, reducing opportunities for positive interactions.

Such interaction patterns can be understood within a dynamic systems framework (Thelen and Smith 2007), as applied to family functioning in family systems theory (Johnson and Ray 2016; Kerr and Bowen 1988; Kozlowska and Hanney 2002; Minuchin 1985) and transactional models of development (Sameroff 2009). Families can be conceptualized as complex adaptive systems in which parents and children continuously influence one another (Granic et al. 2003; Thelen and Smith 2007). Small changes in one component may propagate through the system and influence other aspects of family functioning (Johnson and Ray 2016; Kelledy and Lyons 2019; Trivette et al. 2010). Over time, these interactions can give rise to relatively stable patterns, such as escalating cycles of conflict or reinforcing loops of positive interaction (Johnson and Ray 2016). Accordingly, children's developmental outcomes are shaped not only by individual parenting behaviors but also by the dynamic interaction patterns that emerge within families (Johnson and Ray 2016; Sameroff 2009).

If maladaptive family functioning arises from self‐reinforcing interaction patterns, interventions may be most effective when they directly target these dynamics (Ho et al. 2022; Trivette et al. 2010). Behavioral family interventions aim to improve key aspects of family functioning that shape these processes by targeting parent‐child relationships and parenting behavior (Menting et al. 2013; Nowak and Heinrichs 2008). Consistent with this, positive parenting programs have been shown to reduce dysfunctional parenting practices and improve child outcomes (De Graaf et al. 2008; Kierfeld et al. 2013).

Despite their efficacy (Leijten et al. 2022), most intervention evaluations rely on group‐level (nomothetic) analyses, assuming average effects generalize across families. However, from a dynamic systems perspective, this assumption is problematic, as family interaction patterns emerge from reciprocal processes that may differ substantially across families (Boele et al. 2024). Research at the individual level (idiographic) highlights considerable heterogeneity in family processes, suggesting average effects may obscure meaningful differences in how interactions unfold within families (Boele et al. 2024; Coppersmith et al. 2024; Wang et al. 2024). Examining within‐family dynamics may provide more precise insights into intervention mechanisms (Fosco and Lydon‐Staley 2020; Molenaar 2004). Identifying the mechanisms underlying dysfunctional interaction patterns at the within‐family level may improve intervention acceptability and efficiency by enabling tailored strategies to families' unique interaction dynamics.

### 1.1 Key Variables in Daily Family Interactions

Improved understanding of within‐family interaction dynamics necessitates identifying specific components capturing regulatory mechanisms. Within dynamic family systems, daily interaction patterns are assumed to reflect regulatory processes that either amplify negative exchanges or promote adaptive interactions (Lindblom et al. 2017; Paley and Hajal 2022). Stress‐related escalation processes may trigger conflict (Peltz et al. 2021), whereas positive reinforcement may buffer negative dynamics (Kunnen et al. 2019).

To capture these mechanisms, the present study focuses on four constructs representing key indicators of these processes which are frequently targeted in behavioral parenting interventions: parental stress, parent–child conflict, explicit praise, and shared quality time. The former reflect stress‐related dysregulation processes (Jones et al. 2021), whereas the latter represent positive parental behavior that may counteract escalating interaction cycles (Weeland et al. 2022; Zabidi et al. 2023).

#### 1.1.1 Parental Stress/Conflict

Parental stress is a well‐established predictor of child psychopathology, with longitudinal evidence linking parenting stress to subsequent internalizing and externalizing difficulties in adolescents (De Maat et al. 2021; Dijk et al. 2022). Daily‐level analyses confirm positive associations between parental stress and parent–child conflict (Zabidi et al. 2023), suggesting stress as a proximal trigger of negative interaction cycles and thus representing a key indicator of stress‐related dysregulation within family systems.

#### 1.1.2 Praise/Quality Time

Conversely, supportive parenting behaviors such as explicit praise and shared quality time may function as stabilizing regulatory mechanisms. While praise is often assumed to directly improve child behavior, its effectiveness appears to be context‐dependent (Thomaes and Leijten 2020), optimized when embedded within broader positive attention patterns (Owen et al. 2012; Weeland et al. 2022). Similarly, shared quality time correlates with reduced conflict and better adolescent adjustment (Boele et al. 2020; Zabidi et al. 2023). Together, these behaviors may buffer stress‐related processes and promote adaptive family interaction dynamics.

Beyond observable interaction behaviors, trait individual‐level characteristics may further shape how these processes unfold within families. Within a dynamic systems perspective, individual‐level constructs such as beliefs or traits are shaped through repeated moment‐to‐moment experiences while simultaneously influencing how individuals respond to similar situations in the future. Extending this reasoning to the family context, parental self‐efficacy (PSE), defined as parents' confidence in their ability to manage their child's behavior and development, can be conceptualized as a dynamic regulatory resource embedded within family interaction systems. Drawing on social cognitive theory, parents with higher PSE may be more likely to interpret child misbehavior as manageable and engage in constructive parenting strategies. Accordingly, PSE may shape moment‐to‐moment interaction dynamics in daily family life, for example by buffering the escalation of parental stress into conflict.

Empirical research provides support for this theoretical account. Higher PSE has consistently been associated with lower parenting stress, fewer child behavior problems, and more responsive parenting (Albanese et al. 2019; Fang et al. 2021). Moreover, family interventions have been shown to enhance PSE, improving parental well‐being and parent–child relationships (De Graaf et al. 2008; Mouton and Roskam 2015; Trivette et al. 2010). Despite these findings, the role of PSE in shaping momentary interaction processes in daily family life remains underexplored.

### 1.2 Family‐Specific Network Modeling

These considerations highlight the need for analytical approaches capturing such dynamic, interrelated processes that unfold over time, moving beyond isolated associations and instead modeling the structure of interactions between variables. Network analysis provides a promising framework, conceptualizing psychological variables as elements of interconnected systems that influence one another over time (Borsboom 2017). In doing so, network modeling aligns with dynamic systems perspectives, allowing examinations of how interaction patterns emerge and are maintained within families (Boele et al. 2020, 2024). By enabling the estimation of family‐specific network models, network analysis allows detailed comparisons across families, thereby revealing heterogeneity that is often obscured in group‐level analyses.

Among a range of statistical methods for estimating person‐specific networks (Bringmann 2021; Van Der Gaag 2023), Group Iterative Multiple Model Estimation (GIMME) represents a robust, data‐driven approach capable of simultaneously modeling both idiographic and nomothetic structures (Gates and Molenaar 2012).

GIMME identifies both contemporaneous (within‐day) and lagged (across‐day) associations, demonstrating high accuracy in detecting heterogeneity across idiographic networks (Hoekstra et al. 2023). Notably, GIMME includes a subgrouping feature, identifying data‐driven clusters based on shared network edges not evident at the group level (Lane et al. 2019). From a dynamic systems perspective, such subgroup structures may reflect different regulatory patterns within family interaction systems. Identifying these patterns may therefore provide insights into why families differ in how daily interactions unfold. Given these advantages, GIMME is particularly well‐suited to uncover both shared and family‐specific patterns, providing a nuanced understanding of how daily interactions evolve and relate to PSE.

### 1.3 Present Study

The present study aims to deepen the understanding of heterogeneity in family interactions by investigating daily connections between parental stress, explicit praise, quality time, and family conflict using a family‐specific network approach. These variables were selected because they represent theoretically relevant indicators of regulatory processes within dynamic family systems, capturing both stress‐related escalation (parental stress, conflict) and positive relational reinforcement (praise, quality time). While prior work has found negative associations between quality time and conflict, and positive links between parental stress and conflict (Zabidi et al. 2023), idiographic network structures and temporal dynamics remain uncharacterized. Using daily diary data, this study addresses this gap by analyzing how these variables interact in daily family life through GIMME and whether PSE predicts subgroup membership based on family‐specific network structures.

Our research questions are:

1. *How heterogeneous are family‐specific network structures within and across days?* In line with Zabidi et al. (2023), we hypothesize a negative edge between family conflict and quality time (H1a), and a positive edge between family conflict and parental stress (H1b) in the contemporaneous group‐level network. Limited prior research precludes specific lagged hypotheses, prompting us to adopt an exploratory approach
2. *Is PSE associated with differences in within‐family dynamics?* We hypothesize that PSE predicts subgroup membership based on family‐specific network structures (H2). Specifically, families with higher PSE may exhibit interaction patterns in which positive parenting behaviors buffer stress‐related processes and reduce conflict escalation, whereas families with lower PSE may show stronger links between parental stress and conflict.

## 2 Method

The current study used data from phase three of the AnCu study, a national German online study conducted between February 2021 and June 2022. Preregistration (https://osf.io/waknr) and analyses (https://osf.io/kdh4v/) are publicly accessible, data are available upon request.

### 2.1 Participants

Nonclinical community participants were recruited via social media, study recruitment platforms, and student mailing lists. Only data from the third of three study phases were used for the current analysis. Inclusion criteria required mothers of children aged 6–16 years residing in the same household, with internet access and sufficient German proficiency to complete study materials. In households with multiple eligible children, mothers chose one child to report on. Compensation included a 25€ voucher and lottery eligibility for families participating in earlier study phases.

Of 81 participating mothers, exclusions included: 33 for missing data (≥ 1 week), one for implausible responses, two with children aged ≥ 15 to minimize age‐related confounding (Bodalski et al. 2023), and two with inadequate GIMME model fit (root mean squared error of approximation [RMSEA] ≥ 0.16, standardized root mean residual [SRMR] ≥ 0.13, comparative fit index [CFI] ≤ 0.48, non‐normed fit index [NNFI] ≤ 0.19). The final sample comprised 43 mothers aged 33–53 years (*M* = 41.44 years, SD = 4.61 years) and their children aged 6–14 years (*M* = 9.09 years, SD = 2.35 years; 23 female, 20 male).

### 2.2 Procedure

All procedures were approved by the local ethics committee at Justus‐Liebig University, Giessen (approval number 2021‐0001). The procedures used in this study adhere to the tenets of the Declaration of Helsinki. Informed consent was obtained from all individual participants included in the study.

Before enrolment in the third study phase, telephone screenings confirmed eligibility. Informed consent was obtained for every survey and interview. Trained research assistants administered the Parenting Sense of Competence Scale (PSOC; Johnston and Mash 2012) and a demographic survey during initial interviews. An introductory video explaining the study preceded data collection.

Data collection took place between February and April 2022, comprising a 4‐week baseline assessment followed by a 4‐week behavioral family intervention. Participants completed daily diaries, which were submitted weekly via Unipark (2020). Intervention content, which covered relationship‐building, positive reinforcement, compliance‐promoting instructions, and emotional validation (Gar and Hudson 2009; Garcia‐Lopez et al. 2014), was delivered via two weekly psychoeducational videos with corresponding exercises. Contrary to preregistration specifying baseline‐only analyses, the first intervention week was included to meet the minimum requirement of 30 time points for GIMME.

### 2.3 Measures

PSE was assessed via a german PSOC translation (Johnston and Mash 2012). The PSOC is a self‐assessment scale comprising 16 items rated on a 6‐point Likert scale (*strongly disagree* to *strongly agree*; see Table S1 for all items). Our analyses referred to the total sum score of the PSOC, which ranges from 16 to 96. A higher sum score indicates higher PSE. In line with previous evaluations of its reliability (Oltra‐Benavent et al. 2020), there is good internal consistency for the scale in the present sample (*α* = 0.84). Daily diaries assessed parental stress, parent‐child conflict frequency, explicit praise quantity, and shared quality time duration. Table 1 presents item content and response formats.

**TABLE 1:** Daily diary items to capture daily conflict, quality time, parental stress, and child praise.

Construct	Item	Response format
Conflict	How often did you and your child have a conflict on the specified day in which you argued, shouted at each other, or became physical? It doesn't matter who started the conflict or what it was about	0–3
Conflict	In the case that more than 3 conflicts occurred, please enter the number of conflicts here	Open response
Quality time	How much time did you intentionally spend with your child on the respective day? (This does not include activities like homework or watching TV but rather playing together, cuddling, reading aloud, or having conversations together). (Specify in minutes!)	0–60 min in 5‐min steps
Quality time	In the case that you spent more than 1 h with your child, please enter the number of hours here	Open response
Parental stress	How stressed did you feel on the respective day?	“not at all,” “some‐ what,” “very much”
Praise	How often did you explicitly praise your child on the respective day, for example, by saying that they did something well?	0–6
Praise	In the case that you praised your child more than 6 times, please enter the number of praises here	Open response

### 2.4 Statistical Analysis

All analyses were performed using the software, R (4.4.2) (R Core Team 2024). GIMME is a data‐driven network modeling technique that iteratively builds models using a unified structural equation (uSEM) framework, a form of structural vector autoregressive (VAR) model. It captures temporal and contemporaneous edges at idiographic and nomothetic levels (Gates et al. 2010). Detailed tutorials and algorithm descriptions are provided in prior work (e.g., Gates et al. 2017; Lane et al. 2019). Model selection proceeds hierarchically by fitting individual null models, proceeding to identify a group‐level model (edges that significantly improve model‐fit (*p* < 0.01) for a specified majority of individuals). Individual models are then refined by adding person‐specific edges to achieve optimal model fit (Beltz and Gates 2017). Model fit is deemed sufficient by meeting at least two criteria: RMSEA ≤ 0.05, SRMR ≤ 0.05, CFI ≥ 0.95, and NNFI ≥ 0.95.

S‐GIMME extends this via subgroup detection using Walktrap community detection (Gates et al. 2017), identifying subset‐specific edges absent in the group model. Similar to the group‐level, subgroup edges are added if they significantly improve model fit for a specified subgroup majority (Lane et al. 2019). Unlike traditional multilevel modeling, GIMME minimizes nomothetic effect bias on idiographic estimates (Liu 2017). It addresses missing data using full information maximum likelihood (Beltz and Gates 2017).

In the present study, we modeled contemporaneous and lagged relations among praise (P), parent‐child conflict (C), quality time (QT), and parental stress (S) across individual, subgroup, and group levels. Majority thresholds followed prior studies: 75% for group‐level and 50% for subgroup level (Coppersmith et al. 2024; Yin et al. 2023). The relationship between individual network structures and PSE was planned to be assessed using a multinomial logistic regression, with PSE predicting subgroup membership.

GIMME assumes weak stationarity, that is, constant means, variances and lagged dependence structures of each series over time, as temporal trends in variables may distort parameter estimation. It is further advised to verify the white noise assumption for standardized residuals to confirm temporal dependency removal (Beltz and Gates 2017). Stationarity was evaluated using the augmented Dickey‐Fuller (ADF) and Kwiatkowski‐Phillips‐Schmidt‐Shin (KPSS) test. The white noise assumption was evaluated using the Box‐Pierce test. To improve model reliability, we deviated from preregistration by following a more traditional GIMME approach following simulation studies (Gates et al. 2017), incorporating all autoregressive paths at group level. Given limited guidance on evaluating heterogeneity in idiographic network models, we followed Hoekstra et al. (2023) to assess sample homogeneity. We therefore defined a homogeneity index (HI) as:

$$\mathrm{HI}=100*\frac{\text{ΣGroup}-\text{level edges}}{\Sigma \;\text{Total edges}}$$

where total edges refers to the sum of all group‐ and individual‐level edges.

## 3 Results

### 3.1 Descriptive Statistics

The sample yielded a total of 6039 observations (*M* = 140.4 per dyad, SD = 1.1). Daily explicit praise ranged 0‐10 (*M* = 2.0, SD = 1.4). Daily conflicts ranged 0‐17 (*M* = 0.8, SD = 1.1). Daily quality time ranged 0‐720 min (*M* = 69.1, SD = 83.4). Parental stress ranged 1–3 (*M* = 1.6, SD = 0.7). PSOC total score ranged 51‐91 (*M* = 68.1, SD = 9.6).

### 3.2 Family‐Specific Network Models

All 43 family‐specific network models showed good model fit, *M*_RMSEA_ = 0.01 (SD = 0.02), *M*_SRMR_ = 0.1 (SD = 0.01), *M*_CFI_ = 0.1 (SD = 0.01), *M*_NNFI_ = 1.2 (SD = 0.6). Stationarity testing via ADF and KPSS tests ($\alpha_{\text{Bonf}}$ = 0.0125) yielded contradictory results: ADF test results indicated non‐stationarity in most families (stress: 88%, conflict: 95%, praise: 97%, quality time: 88%), while KPSS testing indicated non‐stationarity in almost no families (stress: 0%, conflict: 2%, praise: 0%, quality time: 0%). Reanalysis excluding week five yielded similar results, ruling out early intervention effects as a source for significant trends. Visual trend inspection indicated no systematic trends (see Figure S1). Given ADF tests' documented risk of inflated false‐negative test results when based on short, autocorrelated time series (DeJong et al. 1992; Schwert 2002) and contrary KPSS and visual inspection results, we assumed weak stationarity.

Box‐Pierce tests ($\alpha_{\text{Bonf}}$ = 0.0125) identified white noise deviations in three variables across three families (family 34, quality time, *χ* ^2^(9) = 22.30, *p* = 0.008; family 40, quality time, *χ* ^2^(9) = 22.28, *p* = 0.008; family 43, praise, *χ* ^2^(9) = 23.47, *p* = 0.005), while 98% residuals likely met white noise assumptions. To further verify model robustness, we reestimated all idiographic models with reversed contemporary edge directionality, comparing global fit indices (RMSEA, SRMR, CFI, NNFI) between the original and the reversed model. Directionality was considered supported when reversed edges led to deterioration in at least two indices. This criterion was met for 89.5% of edges across participants, confirming that the majority of idiographic GIMME models are empirically constrained by the data.

### 3.3 Group‐Level Paths

No significant group‐level associations emerged beyond autoregressive paths, which were automatically included in the model. Descriptively, the most frequent contemporaneous path was between parental stress and conflict, present in 17 families (39.5%), followed by quality time and praise in 14 families (32.6%). Lagged paths were less common, the most frequent being links from conflict to praise and from quality time to conflict, identified in 6 families each (14%). Table 2 summarizes the number of families with significant contemporaneous and lagged paths.

**TABLE 2:** Number of Families with Significant Contemporaneous and Lagged Edges from GIMME.

Outcome	Predictor							
	Conflict		Parental stress		Praise		Quality time	
	+	−	+	−	+	−	+	−
Contemporaneous paths								
Conflict	−	−	−	−	−	−	−	−
Parental stress	17	0	−	−	−	−	−	−
Praise	2	2	1	4	−	−	−	−
Quality time	6	4	1	6	14	0	−	−
Lagged paths								
Conflict	(28)	(15)	2	1	0	2	3	3
Parental stress	3	1	(32)	(11)	0	3	1	1
Praise	6	0	2	3	(35)	(8)	1	4
Quality time	1	0	3	2	2	2	(33)	(10)

*Note:* All values are based on GIMME analyses across 43 families. + indicates a positive edge, − indicates a negative edge between variables. Contemporaneous edges do not allow conclusions about the direction of the relationship between variables. Lagged edges reflect the next‐day effect of the predictor variable on the outcome variable. Lagged edges in parentheses indicate autoregressive edges.

Individual network models showed high heterogeneity. HI for lagged paths was 25% (four group‐level, 16 total edges), 0% for contemporaneous paths (0 group‐level, 12 total edges). Although edges were present in most families, their number, directionality, and timescale varied widely and were highly family‐specific.

### 3.4 Subgroup‐Level Paths

Two subgroups were identified (*N*_Subgroup 1_ = 7, *N*_Subgroup 2_ = 8). Subgroup stability evaluation via comparison of modularity values from the original similarity matrix to increasingly perturbed matrices (Gates and Fisher 2019) revealed limited robustness (*Q*_0.95_ = 0.57, *Q*_orig_ = 0.21). Neither subgroup exhibited significant subgroup‐level edges. As Wright et al. (2019) note, GIMME subgroups may form from similarities in group‐level estimates without shared edges. Post‐hoc comparisons of subgroup differences in beta estimates of group‐level edges, PSOC scores, and average daily variable levels showed no significant effects (see Table S2). Given low robustness and absence of significant edges, we refrain from further interpreting the identified subgroups.

### 3.5 Exemplary Family‐Specific Network Models

A selection of family‐specific network model visualizations is presented in Figure 1. Their heterogeneity is reflected in the diversity of temporal dynamics, directionality, and polarity. For instance, praising the child more predicted less parental stress on the following day in family A, which predicted more quality time on the third day. For family B, no significant effects from one day onto the next were present. On a contemporaneous timescale, more quality time was associated with less conflicts. Contrary to family B, family C experienced more conflicts on days with more quality time. In this family, more explicit praise predicted less conflicts and more quality time on the following day. All 43 network models, model fit indices and path estimates can be viewed on https://osf.io/kdh4v/.

**FIGURE 1:**
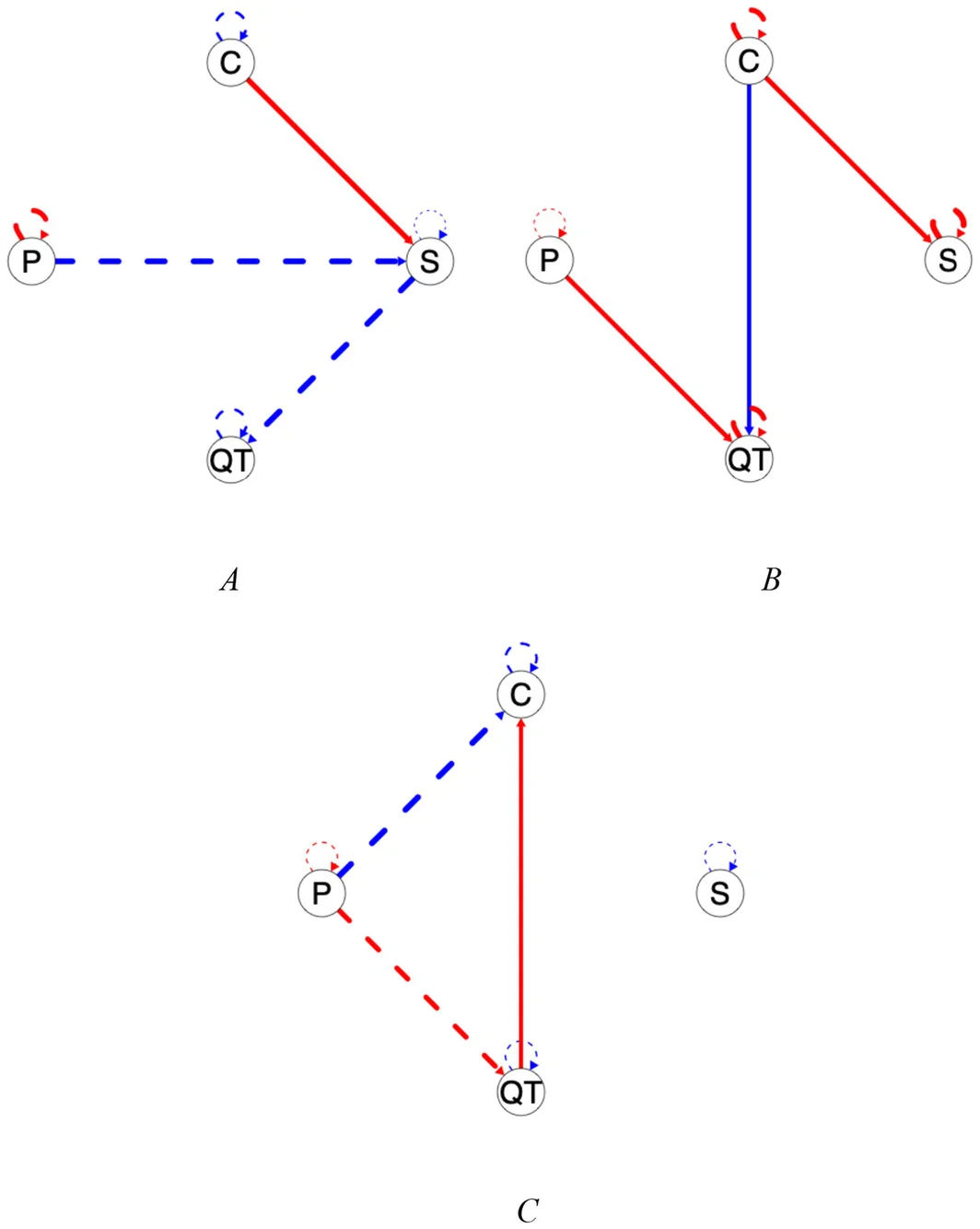
Visual network models of three exemplary families created with GIMME. C, conflict; S, parental stress; QT, quality time; P, praise; red, positive paths; blue, negative paths; solid lines, contemporaneous paths; dashed lines, lagged paths.

### 3.6 Post Hoc PSE Analyses

As GIMME did not yield meaningful subgroups, we deviated from the preregistered plan to regress subgroup membership on PSOC scores. All deviations from the preregistered analytic strategy are documented in Table S3. To explore whether PSE as a dynamic regulatory resource embedded within family interaction systems might moderate daily interaction patterns, we conducted post hoc analyses. Confirmatory subgroups were defined prior to GIMME estimation using two approaches: (a) quantile‐based PSOC splits into four groups (*PSOC*_Group 1_ = [51–61], *PSOC*_Group 2_ = [62–69], *PSOC*_Group 3_ = [70–73], *PSOC*_Group 4_ = [74–91]), and (b) a median split (*Mdn* = 69.0). Neither approach revealed significant subgroup‐level paths. Additional Spearman's rank correlations between PSOC scores and mean daily levels of conflict, praise, parental stress, and quality time were nonsignificant (see Table S4).

## 4 Discussion

The present study examined heterogeneity in daily family interaction dynamics by analyzing associations between parental stress, explicit praise, quality time, and parent–child conflict applying family‐specific network modeling. Drawing on a dynamic systems perspective, these variables were conceptualized as indicators of regulatory processes capturing stress‐related escalation and positive relational reinforcement. Daily diary data from 43 mother‐child dyads yielded group‐, subgroup‐ and individual‐level network models. We found substantial heterogeneity in family‐specific network structures, with high variability in path presence, directionality and polarity. Contrary to hypotheses H1a and H1b, no contemporaneous or lagged paths achieved the 75% threshold for group‐level inclusion. We also examined whether PSE, conceptualized as a potential regulatory resource, predicted network structures. Contrary to H2, PSE was not significantly linked to subgroup membership or daily interaction variables.

### 4.1 Heterogeneity in Daily Family Interactions

Our findings suggest that daily family interaction dynamics do not follow universal patterns across families. From a dynamic systems perspective, this heterogeneity is not surprising: Interaction patterns emerge from reciprocal feedback processes that may differ across families in structure, strength, and direction (Boele et al. 2020, 2024). The absence of group‐level paths therefore does not necessarily imply an absence of meaningful dynamics. At the same time, given the limited number of observations available for each family, the estimated variability across family‐specific networks should be interpreted cautiously. Some apparent differences in path presence, direction, or strength may reflect estimation uncertainty rather than stable differences in the underlying family processes. Nevertheless, nearly all family‐specific networks included at least one path, suggesting that potentially meaningful regulatory processes were present, although their organization appeared to differ across families. This interpretation aligns with idiographic research showing that group‐level estimates may obscure substantial heterogeneity in daily family functioning (Boele et al. 2020, 2024; Coppersmith et al. 2024; Wang et al. 2024).

Although no universal paths emerged, some associations were relatively common. In 39.5% of families, higher parental stress coincided with increased conflict, consistent with the idea of stress functioning as a proximal dysregulation indicator (Zabidi et al. 2023). In 32.6% of families, praise and quality time were positively linked, suggesting co‐occurrence as positive reinforcement. However, conflict‐quality time associations varied directionally across families. For some families, shared time coincided with less conflict; for others, with more conflict. This illustrates that behaviors may serve different functions depending on broader family system dynamics. Similarly, praise may reflect positive reinforcement in some families but repair attempts after conflict in others, indicating context‐dependent regulatory functions. The predominance of contemporaneous over lagged associations suggests that many regulatory processes unfold within relatively short time windows rather than persisting across days. Once‐daily assessments may therefore be too coarse to capture faster feedback processes occurring within hours or specific interaction episodes.

### 4.2 PSE and Family‐Specific Network Structures

The current study did not support the hypothesis that PSE is associated with family‐specific network structures. This finding is noteworthy given our theoretical assumption that PSE may operate as a regulatory resource within dynamic family systems. Neither PSE‐based subgroup modeling nor correlational analyses revealed significant associations with daily conflict, praise, parental stress, or quality time, contrasting sharply with prior research consistently linking higher PSE to reduced parenting stress and more promotive parenting behaviors (e.g., Albanese et al. 2019; Fang et al. 2021).

One explanation may be that PSE was measured as a relatively stable self‐belief, whereas family interactions were assessed as short‐term daily processes. From a dynamic systems perspective, such characteristics may both shape and be shaped by repeated moment‐to‐moment experiences (Johnson and Ray 2016), but these reciprocal influences may not be detectable when PSE is assessed only once. Future studies should examine daily or momentary PSE fluctuations to test whether state‐like changes relate more closely to family interaction dynamics. Moreover, heterogeneity in family‐specific networks may also reflect other unmeasured system components, such as child temperament, parental emotion regulation, relationship quality, daily stressors, or broader contextual demands. PSE may therefore represent only one of several factors that shape how daily interaction dynamics unfold within families. Finally, methodological constraints, including limited timepoints and high heterogeneity, may have further reduced subgroup detection capacities (Mansueto et al. 2023).

### 4.3 Limitations and Future Outlook

This study is the first applying idiographic temporal network analysis to daily family interactions regarding parental stress, praise, quality time, and conflict. Conducted in a community sample in a non‐laboratory setting, its design enhanced ecological validity and reduced retrospective bias.

Nevertheless, several limitations warrant discussion. Including only mothers limits generalizability of our findings, excluding children's, fathers' and nonbinary parents' perspectives. Additionally, all daily variables relied on parent report, meaning networks may partly reflect parents' subjective perceptions rather than dyadic processes. Shared method variance and parent‐specific interpretation biases may have influenced estimations. Future studies should include child reports, observational data, or multi‐informant designs.

Second, the number of daily observations per family was low and only just met the minimum requirements applied for GIMME, limiting the precision and stability of the family‐specific network estimates. Weaker associations may have remained undetected, while identified paths and between‐family differences may partly reflect sampling variability. Although prior simulation work does not suggest that GIMME generally produces inflated false‐positive rates (Nestler and Humberg 2021), absent paths should not be interpreted as evidence that a process is irrelevant, and the observed heterogeneity and subgroup structures should be considered preliminary. As established reliability methods for idiographic temporal networks remain limited, replication with longer time series and sensitivity analyses is needed to distinguish robust dynamics from statistical noise. Lastly, focusing on only four aspects of family interaction to increase network stability likely oversimplified the complexity of daily family life.

Despite these limitations, the present study has important implications for family research and interventions. Rather than questioning the overall utility of behavioral family interventions, our findings suggest that the mechanisms through which such interventions operate may differ across families. From a dynamic systems perspective, this implies that similar intervention components may affect different feedback processes depending on the family‐specific organization of daily interactions. These results resonate with developments in psychopathology research, where idiographic network models have informed person‐specific therapeutic interventions (e.g., Andreasson et al. 2023; Klintwall et al. 2023). To our knowledge, network‐informed approaches have not yet been systematically extended to family interventions. The present findings suggest that family‐specific network models could help identify which interaction patterns maintain dysfunctional dynamics in a given family and thereby guide the selection of intervention components. Such an approach could improve the efficiency and acceptability of family interventions by aligning treatment strategies with families' unique regulatory patterns. Given the limited number of observations per family, the clinical implications remain preliminary. The present networks should not yet guide individual treatment decisions but may inform the development and prospective evaluation of network‐informed family interventions. Future research should therefore examine whether network‐informed intervention planning can support more tailored and mechanism‐based family interventions.

## 5 Conclusion

In conclusion, the present study provides evidence for substantial heterogeneity in daily family interaction dynamics. Associations between parental stress, praise, quality time, and parent–child conflict differed markedly across families, suggesting that stress‐related dysregulation and positive relational reinforcement may be organized differently within family systems. From a dynamic systems perspective, these findings support the view that family functioning emerges from family‐specific interaction patterns rather than universal processes. Contrary to expectations, PSE was not associated with subgroup membership or daily interaction dynamics, possibly reflecting the complexity of its role as a dynamic regulatory resource within families. Overall, the findings highlight the potential value of family‐specific network models for identifying mechanisms that maintain dysfunctional interaction patterns and for informing more tailored, mechanism‐based family interventions.

## Funding

The authors have nothing to report.

## Conflicts of Interest

The authors declare no conflicts of interest.

## Supporting information


**Table S1:** Parenting Sense of Competence Scale items with their German Translation, Scoring and Anchors.
**Table S2:** Results of difference testing between Subgroup 1 and Subgroup 2.
**Table S3:** Overview of deviations from the preregistered analytic approach.
**Table S4:** Spearman's rank correlations between PSOC total score and mean conflict, praise, parental stress, and quality time.
**Figure S1:** Time series plots for parental stress, parent–child conflict, explicit praise, and shared quality time across all participants.
