# Supplementary material for: Exploring Family Interactions: Heterogeneity in Family Specific Network Models
Source: Fam Process. 2026 Oct 9;65(4):e70208. doi: 10.1111/famp.70208 (PMC13652705; doi:10.1111/famp.70208)
Supplement: Supplementary file 1 [file FAMP-65-0-s001.docx]

**Supplemental Materials**

# Table S1

*Parenting Sense of Competence Scale Items with their German Translation, Scoring and Anchors*

| **Item** | **German Translation** | **Score** | **Anchor** |
| --- | --- | --- | --- |
| The problems of taking care of a child are easy to solve once you know how your actions affect your child, an understanding I have acquired. | Wenn man weiß, wie das eigene Handeln auf das Kind wirkt, lassen sich alle Erziehungsprobleme leicht lösen. | 1 to 6 | *strongly disagree* to *strongly agree* |
| Even though being a parent could be rewarding, I am frustrated now while my child is at his / her present age. | Mutter/Vater zu sein ist zwar schön, aber im Moment macht mir das Alter, in dem mein Kind sich befindet, zu schaffen. | 1 to 6 | *strongly disagree* to *strongly agree* |
| I got to bed same way I wake up in the morning, feeling I have not accomplished a whole lot. | Am Ende eines Tages habe ich oft das Gefühl nicht viel erreicht zu haben | 1 to 6 | *strongly disagree* to *strongly agree* |
| I do not know why it is, but sometimes when I’m supposed to be in control, I feel more like the one being manipulated. | Ich weiß nicht, woran es liegt, aber manchmal habe ich das Gefühl, ich werde manipuliert, obwohl ich eigentlich Kontrolle ausüben sollte. | 1 to 6 | *strongly disagree* to *strongly agree* |
| My mother/ my father was better prepared to be a good parent than I am. | Meine Mutter/mein Vater war besser vorbereitet, eine gute Mutter/ein guter Vater zu sein, als ich es bin. | 1 to 6 | *strongly disagree* to *strongly agree* |
| I would make a fine model for a new parent to follow in order to learn what they would need to know in order to be a good parent. | Ich könnte ein gutes Vorbild für angehende Mütter/ Väter sein. | 1 to 6 | *strongly disagree* to *strongly agree* |
| Being a parent is manageable, and any problems are easily solved. | Probleme, die sich einer Mutter/einem Vater stellen, lassen sich in der Regel lösen. | 1 to 6 | *strongly disagree* to *strongly agree* |
| A difficult problem in being a parent is not knowing whether you’re doing a good job or a bad one. | Mutter/Vater sein ist deshalb so schwierig, weil man nicht weiß, ob man es gut macht oder nicht. | 1 to 6 | *strongly disagree* to *strongly agree* |
| Sometimes I feel like I’m not getting anything done. | Manchmal habe ich das Gefühl, nichts auf die Reihe zu bekommen. | 1 to 6 | *strongly disagree* to *strongly agree* |
| I meet my own personal expectations for expertise in caring for my child. | Was die Erziehung meines Kindes angeht, erfülle ich meine eigenen Erwartungen. | 1 to 6 | *strongly disagree* to *strongly agree* |
| If anyone can find the answer to what is troubling my child, I am the one. | Wenn irgendjemand herausfinden kann, was mein Kind bedrückt, dann bin ich es. | 1 to 6 | *strongly disagree* to *strongly agree* |
| My talents and interests are in other areas, not being a parent. | Meine Stärken und Interessen liegen in anderen Bereichen, und nicht darin, eine gute Mutter/ein guter Vater zu sein. | 1 to 6 | *strongly disagree* to *strongly agree* |
| Considering how long I’ve been a parent, I feel thoroughly familiar with this role. | Wenn ich darüber nachdenke, wie lange ich schon Mutter/ Vater bin, dann fühle ich mich sehr vertraut in dieser Rolle. | 1 to 6 | *strongly disagree* to *strongly agree* |
| If being a parent of a child were only more interesting, I would be motivated to do a better job as a parent. | Wenn Mutter/Vater sein eine interessantere Aufgabe wäre, dann würde ich mich mehr anstrengen, eine bessere Mutter/ein besserer Vater zu sein. | 1 to 6 | *strongly disagree* to *strongly agree* |
| I honestly believe I have all the skills necessary to be a good parent to my child. | Ich glaube, dass ich alle notwendigen Fähigkeiten einer guten Mutter/eines  guten Vaters besitze. | 1 to 6 | *strongly disagree* to *strongly agree* |
| Being a parent makes me tense and anxious. | Mutter/Vater zu sein macht mich nervös und ungeduldig | 1 to 6 | *strongly disagree* to *strongly agree* |

# Table S2

*Results of Difference Testing between Subgroup 1 and Subgroup 2*

# Parameter Subgroup 1 Subgroup 2 *t*(df) *df p*_bonf_

|  | *M* | *SD* | *M* | *SD* |  |  |  |
| --- | --- | --- | --- | --- | --- | --- | --- |
| C ∼ Clag Beta estimate | 0.1 | 0.3 | 0.2 | 0.2 | -0.92 | 11.5 | 1.00 |
| P ∼ Plag Beta estimate | 0.3 | 0.2 | 0.4 | 0.2 | -0.37 | 12.2 | 1.00 |
| QT ∼ QTlag Beta estimate | 0.3 | 0.2 | 0.6 | 0.2 | -2.63 | 12.7 | .21 |
| S ∼ Slag Beta estimate | 0.4 | 0.3 | 0.3 | 0.2 | 0.66 | 11.2 | 1.00 |
| Daily conflict | 0.9 | 0.9 | 0.8 | 0.5 | 0.07 | 8.7 | 1.00 |
| Daily praise | 2.1 | 0.9 | 2.1 | 1.0 | -0.05 | 13.0 | 1.00 |
| Daily quality time | 65.4 | 44.0 | 92.7 | 35.1 | -1.32 | 11.5 | 1.00 |
| Daily parental stress | 1.7 | 0.4 | 1.5 | 0.3 | 1.23 | 12.4 | 1.00 |
| PSOC score | 73.4 | 11.5 | 65.1 | 5.6 | 1.75 | 8.4 | 1.00 |

*Note.* Mean parameter values for each of the analyses are shown for subgroup 1 (*n* = 7) and subgroup 2 (*n* = 8), as well as the results of *t* tests (assuming unequal variance) with Bonferroni-adjusted p-values comparing the parameter values between the two groups. X ∼ Xlag Beta estimates indicates autoregressive path estimates for daily conflict (C), daily praise (P), daily quality time (QT), and daily parental stress (S).

# Table S3

*Overview of Deviations from the Preregistered Analytic Approach*

| **Preregistration** | **Analytic approach** |
| --- | --- |
| Using 4 weeks of daily diary data. | Additionally including the 5th week of daily diary data (1st week of family intervention phase) in order to meet minimum time point requirements for GIMME. |
| Multinomial logistic regression with the model *subgroup* ∼ *PSE* to test the hypothesis ”PSE is a significant predictor of the subgroup an individual is placed in based on their network structure.” | 1. 1. S-GIMME analysis with predefined subgroups.    - Four subgroups based on quantile-based splits for PSOC scores    - Two subgroups based on median-split for PSOC scores   2. Spearman’s rank correlation between PSOC and mean daily conflict, mean daily praise, mean daily parental stress and mean daily quality time. |
| Stationarity testing via augmented Dickey-Fuller testing. | Stationarity testing via augmented Dickey-Fuller testing, Kwiatkowski-Phillips-Schmidt-Shin testing and visual trend inspection. |
| No additional assessment of network stability. | Re-estimation of all idiographic models with reversed contemporary edge directionality, comparing global fit indices (RMSEA, SRMR, CFI, NNFI) between the original and the reversed model. |
| Exploring the network structure of the temporal group-level network by qualitatively assessing the in-degree and out-degree of every node in the network. | No significant paths in the group-level network were identified, hence the in-degree and out-degree of the nodes was not assessed. |
| No additional assessment of network stability. | Re-estimation of all idiographic models with reversed contemporary edge directionality, comparing global fit indices (RMSEA, SRMR, CFI, NNFI) between the original and the reversed model. |

# Table S4

*Spearman’s Rank Correlations Between* *PSOC Total Score and Mean Conflict,* *Praise, Parental Stress, and Quality Time*

| **Variables** | *r_s_***(41)** | *p***bonf** |
| --- | --- | --- |
| PSOC & Conflict | - .22 | .628 |
| PSOC & Praise | .11 | 1.00 |
| PSOC & Parental Stress | - .26 | .372 |
| PSOC & Quality Time | - .05 | 1.00 |

*Note. r_s_* = Spearman’s rank correlation; *p*_bonf_ = the Bonferroni-corrected p-value.

# Figure S1

*Time Series Plots for Parental Stress, Parent-child Conflict, Explicit Praise, and Shared Quality Time across All Participants*

*Note.* Each panel displays observed daily values (solid lines), linear trend estimates (dashed lines), and LOESS smoothing curves (blue lines). S = Parental Stress; C = Parent-child Conflict; P = Explicit Praise; QT = Shared Quality Time. LOESS = locally estimated scatterplot smoothing with span = 0.75. Y-axes are scaled individually per variable.
